# Tuberous Sclerosis Complex as Disease Model for Investigating mTOR-Related Gliopathy During Epileptogenesis

**DOI:** 10.3389/fneur.2020.01028

**Published:** 2020-09-17

**Authors:** Till S. Zimmer, Diede W. M. Broekaart, Victoria-Elisabeth Gruber, Erwin A. van Vliet, Angelika Mühlebner, Eleonora Aronica

**Affiliations:** ^1^Department of (Neuro)Pathology, Amsterdam Neuroscience, Amsterdam University Medical Centers, University of Amsterdam, Amsterdam, Netherlands; ^2^Department of Pediatrics and Adolescent Medicine, Medical University of Vienna, Vienna, Austria; ^3^Swammerdam Institute for Life Sciences, Center for Neuroscience, University of Amsterdam, Amsterdam, Netherlands; ^4^Stichting Epilepsie Instellingen Nederland (SEIN), Heemstede, Netherlands

**Keywords:** tuberous sclerosis (TSC), mammalian target of rapamycin (mTOR), epilepsy, astrocyte, microglia, oligodendrocyte, glia, epileptogenesis

## Abstract

Tuberous sclerosis complex (TSC) represents the prototypic monogenic disorder of the mammalian target of rapamycin (mTOR) pathway dysregulation. It provides the rational mechanistic basis of a direct link between gene mutation and brain pathology (structural and functional abnormalities) associated with a complex clinical phenotype including epilepsy, autism, and intellectual disability. So far, research conducted in TSC has been largely neuron-oriented. However, the neuropathological hallmarks of TSC and other malformations of cortical development also include major morphological and functional changes in glial cells involving astrocytes, oligodendrocytes, NG2 glia, and microglia. These cells and their interglial crosstalk may offer new insights into the common neurobiological mechanisms underlying epilepsy and the complex cognitive and behavioral comorbidities that are characteristic of the spectrum of mTOR-associated neurodevelopmental disorders. This review will focus on the role of glial dysfunction, the interaction between glia related to mTOR hyperactivity, and its contribution to epileptogenesis in TSC. Moreover, we will discuss how understanding glial abnormalities in TSC might give valuable insight into the pathophysiological mechanisms that could help to develop novel therapeutic approaches for TSC or other pathologies characterized by glial dysfunction and acquired mTOR hyperactivation.

## Introduction

Tuberous sclerosis complex (TSC) is a rare, genetic multisystem disorder with a prevalence of ~1:6,000 newborns. Common symptoms in TSC include benign tumor growth in kidney, heart, lung, eyes, skin, and brain (1). Characteristic lesions in the brain are cortical tubers and ventricular subependymal nodules, which may progress into subependymal giant cell astrocytomas (SEGAs) (2–4). Neurological manifestations include epilepsy, neurodevelopmental delay, and TSC-associated neuropsychiatric disorders (TANDs), such as intellectual disability and autism spectrum disorder (ASD) (5–7). Moreover, as one of the most debilitating symptoms, TSC represents the most common genetic cause for pediatric epilepsy, with roughly 85% of cases developing seizures, predominantly within the first year of life, and 60% eventually presenting with refractory epilepsy (8, 9). Because uncontrolled seizure activity aggravates cognitive comorbidities, immediate seizure management after or ideally before epilepsy onset is crucial for normal cognitive development of patients (10–12). Currently, the most effective long-term treatment for epilepsy in TSC is vigabatrin, a highly effective drug against infantile spasms in TSC patients (13–15), whereas a subgroup of eligible patients benefits from adjunctive everolimus [mammalian target of rapamycin (mTOR) inhibitor] treatment or surgical resection of the suspected epileptogenic lesion (14, 16–19).

TSC is caused by loss-of-function mutations in the tumor suppressors *TSC1* or *TSC2*, both of which are negative regulators of the mTOR (20, 21). Purely heterozygous germline mutations, as well as mosaic mutations, have been detected in TSC patients (21, 22). mTOR is a serine/threonine protein kinase and the catalytic subunit of mTOR complex 1 (mTORC1) and mTORC2. Under normal conditions, mTOR activity is tightly controlled by upstream regulators and acts as important sensor of cellular energy status and homeostasis. Environmental stimuli, such as cytokines or growth factors can stimulate mTOR, enabling cells to dynamically respond to various extracellular cues via adaptation in metabolism or cellular growth (23, 24). Mutations in either *TSC1* or *TSC2* lead to uncoupling from upstream regulators and abnormal hyperactivation of mTORC1, causing growth of the characteristic lesions during brain development. While TSC represents the prototypic monogenic disorder of mTOR hyperactivation, other malformations of cortical development, such as megalencephaly, hemimegalencephaly, and focal cortical dysplasia (FCD) are also characterized by aberrant mTOR activation due to acquired mutations in various mTOR regulators (25). Importantly, all share histopathological and clinical characteristics with TSC; hence, this spectrum of diseases is collectively referred to as mTORopathies [reviewed in (26, 27)].

Importantly, mTOR hyperactivity seems to be directly linked to epileptogenesis as mTOR inhibitors can suppress seizures in preclinical TSC models (28, 29), as well as in clinical studies aimed at treating TSC and SEGAs (16–19, 30, 31). Current consensus is that mTOR inhibitors induce a temporary anticonvulsant effect as do currently available antiepileptic drugs, but may also possess disease-modifying potential (15, 32). The clear causative role of mTOR as epileptogenic driver, as well as implications of mTOR activation in acquired epilepsies (33–36), makes TSC an attractive disease model to utilize as translational prototype for epilepsy in general. Despite the progress in understanding the role of the mTOR signaling pathway, there is still a lack in pinpointing the precise cellular substrates responsible for producing seizures. Interestingly, although the neuropathological hallmarks of TSC are primarily found in tubers, some studies showed that the seizure focus in TSC brains could also originate from the surrounding normal-appearing cortex, based on seizure freedom after resection of the perituberal zone, tissue analysis, and electrocorticographic recordings (37–40). However, further progress in the careful examination and advances in the identification of novel histopathological markers may make a discrimination between tuber and perituber obsolete, eventually. Nevertheless, surgical resection of the tuber leads to seizure relief in 50% to 60% of cases, suggesting an important role in epileptogenesis in at least a subset of patients with a clear-cut epileptogenic “driver” lesion (41–44).

**Figure 1:**
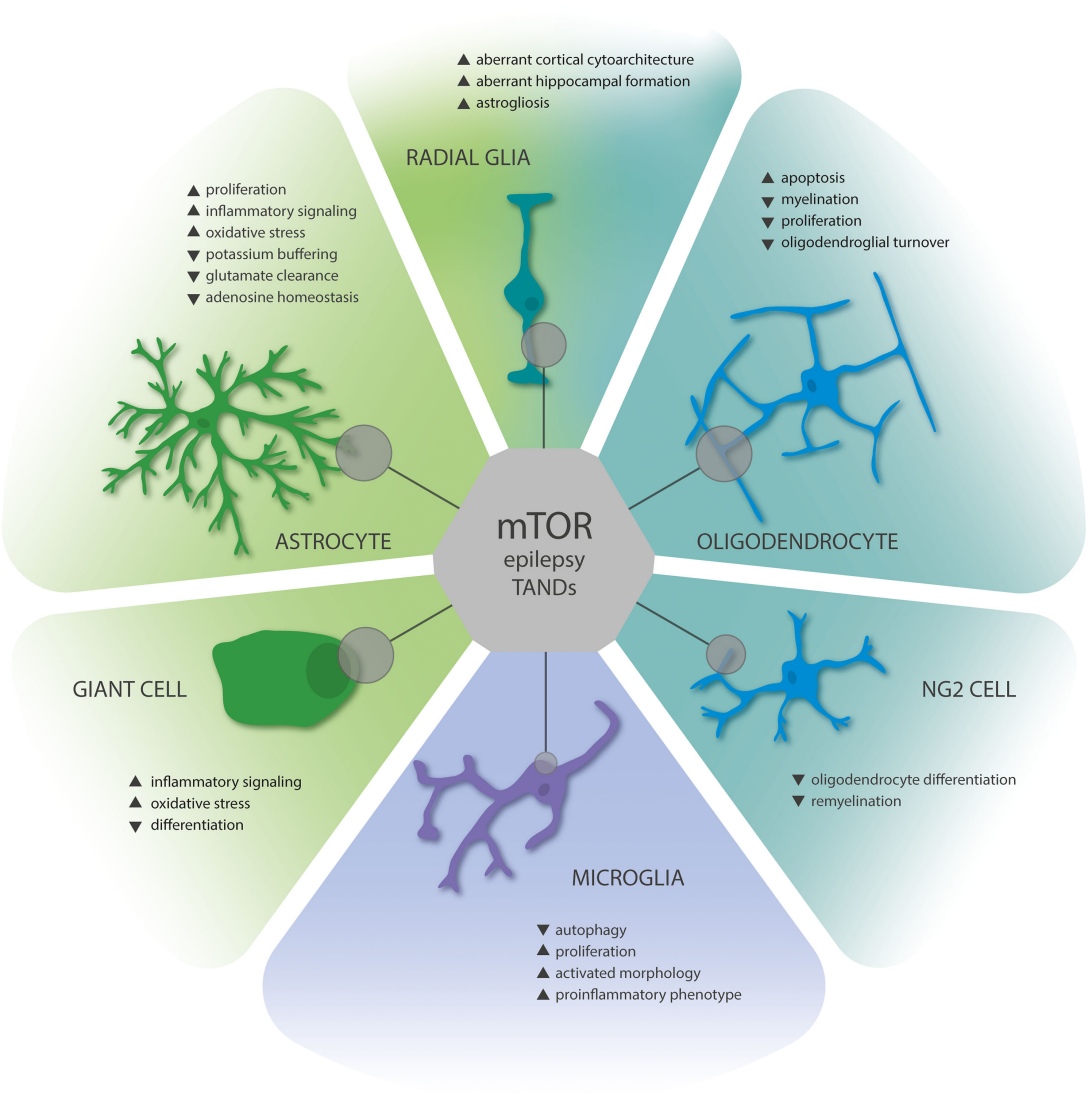
Summary of gliopathic changes due to mTOR hyperactivation in TSC brain lesions. Astrocytes display increased proliferation, activation, and enhanced expression of proinflammatory mediators. Moreover, astrocytes are characterized by decreased homeostatic functions related to ion homeostasis and neurotransmitter metabolism. Radial glia, the neuroglial precursors of astrocytes, oligodendrocytes, and neurons, contribute to malformations of cortical development and aberrant gliogenesis, as well as the formation of giant cells, which display characteristics of proinflammatory glia. Oligodendrocyte dysfunction leads to hypomyelination and disturbed remyelination, and their proliferation is reduced. While dysfunction of NG2 glia in TSC deserves further investigation, they are crucially involved in myelination and crosstalk with neurons, thus representing an essential component of TSC gliopathology. Finally, as for astrocytes, microglia are characterized by enhanced proliferation, activation, and expression of proinflammatory mediators; however, these changes are likely secondary to mTOR activation in the TSC brain. Collectively, these changes contribute to epilepsy and neuropsychiatric comorbidities in TSC. The influence of mTOR signaling on the individual cell types is indicated by the size of gray circles.

In the brain of TSC patients, mTOR hyperactivity promotes development of often multifocal brain lesions characterized by aberrant glioneuronal proliferation, cortical dyslamination, and hypomyelination, along with the presence of dysplastic neurons and improperly developed giant cells (4, 27, 45–47). TSC tissue obtained from surgery due to refractory epilepsy usually presents with a heterogeneous frequency of the aforementioned histopathological hallmarks between patients (27, 46). TSC lesions are thought to arise by the Knudson hypothesis, also known as the “two-hit” hypothesis (48). Accordingly, somatic mutations in either *TSC1* or *TSC2*, resulting in the loss of wild-type alleles, have been detected in different types of TSC neoplastic lesions and to a lesser extent in cortical tubers (21, 49, 50). Thus, it is still an ongoing matter of discussion whether monoallelic inactivation of TSC1/TSC2 is sufficient for tuber development or if the second hit occurs in a specific cellular component complicating its identification (21, 49, 50). Cell specificity, mutation load, and mutation timing during brain development likely give rise to the diverse neuropathological presentations. Recent evidence from *in vitro* cell cultures and organoid models of TSC revealed that mTORC1 activity during cortical development is tightly controlled, and mTORC1 suppression is required for proper neurogenesis (51). Of note, mTORC1 hyperactivity promotes gliogenesis, likely explaining the increased number of glia in tubers (52–56). More specifically, mTORC1 was shown to activate STAT3 signaling, which represents a major driver of gliogenesis during development (53, 57–60) Furthermore, gliosis and activation of inflammatory signaling pathways are histopathological hallmarks of TSC (46, 52, 61–64). Accordingly, although dysfunctional neuronal circuitry is ultimately required for the development of epilepsy and mTOR can directly regulate neuronal structure, function, and plasticity (65–68), accumulating evidence shows that glial cells represent a crucial element in the pathogenesis of TSC and might pose novel therapeutic strategies (64). This review will focus on the role of glial dysfunction related to mTOR hyperactivity and its contribution to comorbidities, such as epilepsy and TANDs in TSC. In this context, while many studies primarily focused on neuroglial crosstalk, we will emphasize aberrant interglial communication as an essential aspect of TSC. Finally, studying glial abnormalities in TSC might give valuable insight into pathophysiological mechanisms, which could help to develop novel therapeutic approaches for TSC or other pathologies characterized by gliopathic changes and acquired mTOR hyperactivation (summarized in Figure 1).

## Astrocytes

Astrocytes display distinct functional changes in a variety of epilepsies with different etiologies, and it becomes increasingly clear that they play crucial roles in the process of epileptogenesis, including TSC (69, 70). Neuropathological hallmarks in resected cortical tubers of TSC patients include increased expression of glial fibrillary acidic protein (GFAP), vimentin, and S100β, as well as higher numbers of astrocytes. Moreover, these astrocytes often present dysplastic and reactive phenotypes compared to the perituberal area and control brain tissue (71, 72). While most studies characterize the total population of astrocytes, some report different subpopulations of astrocytes in TSC (52, 72). One study characterized two subpopulations of astrocytes: “reactive” cells, which are large and vimentin positive and reveal mTOR activation, and “gliotic” astrocytes, which are smaller, do not show mTOR activation, and resemble gliotic astrocytes found in hippocampal sclerosis (HS) (52). Gliotic astrocytes, as in HS, present with decreased expression of inwardly rectifying potassium (Kir) channel subunit Kir4.1, a decrease in the glutamate transporters excitatory amino acid transporter 1 (EAAT1) and EAAT2, and a decrease in glutamine synthetase, all of which represent proepileptogenic changes (70). The authors of this study concluded that the gradual transformation from reactive to gliotic astrocytes might represent a major driving force for the morphological dynamics of tubers over time (52). Another study discriminated between normal astrocytes (no mTOR activation, vimentin-negative, and GFAP-positive), reactive astrocytes (no mTOR activation, vimentin-positive, and GFAP-positive), and dysplastic astroglia (mTOR activation, vimentin-positive, and GFAP-negative), the latter representing an expression pattern common to immature astrocytes and radial glia (72). Taken together, both studies support the notion that populations of improperly differentiated astrocytes with mTOR activation, as well as properly developed, reactive astrocytes without mTOR activation, contribute to TSC pathology. Here, the aforementioned continuum of pathological changes in astrocytes and the precise cellular composition of the tissue might reflect the intrinsic epileptogenicity of the tuber. Importantly, the functional changes in TSC astrocytes are likely caused by a combination of the reactive state in response to seizures known from other diseases, such as mesial temporal lobe epilepsy (TLE), which could induce secondary mTOR activation (33), but also general disturbance in protein translation caused by sustained mTOR activation in mutation-carrying cells. Ultimately, both astrocyte subpopulations could end up having different pathogenic origins, but similar functional outcomes in terms of expression of Kirs, EAATs, or glutamine synthetase, further increasing the epileptogenic potential of the tuber. Whether the different degrees of mTOR activation underlie the wide diversity of astrocyte functions and phenotypes in TSC deserves further investigation. However, for neurons, it has already been shown that extent of mTOR hyperactivity correlates with seizure severity and associated neuropathology (73). Finally, in addition to intrinsic astrocytic properties, maintenance of a non-reactive state in astrocytes was also shown to depend on neuronal mTORC1 signaling, adding yet another level to altered astrocyte function in TSC (74).

The most striking evidence for astrocytic contribution to epileptogenesis in TSC comes from a conditional *Tsc1* knockout mouse model (referred to as *Tsc1*^GFAP^ mice), in which *Tsc1* is specifically deleted in GFAP-expressing cells during embryonic development, leading to mTOR hyperactivity in these cells (75, 76). Notably, *Tsc1* deletion is also induced in GFAP-positive neural progenitor cells and can be found in neurons, thereby blurring the specific contribution of astrocytes to some extent (77). While this model does not recapitulate all pathological hallmarks of human TSC (most notably lacking tuber formation and giant cells), development of spontaneous recurrent seizures arises in all animals at 1 month of age. This occurs likely via diffuse astrocyte proliferation and dispersion of neurons, causing altered neuronal circuitry. Interestingly, even post-natal deletion of *Tsc1* at 2 weeks of age leads to development of epilepsy in half of the animals, although in a less severe form (77). Consequently, *TSC1* deletion appears to be the initial insult followed by a latent stage of epileptogenesis, which in TSC patients might be even prenatally. Notably, treatment of *Tsc1*^GFAP^ mice with the mTOR inhibitor rapamycin suppressed seizures, whereas vigabatrin reduced seizures and partially inhibited mTOR activity, astrogliosis, and neuronal disorganization (29, 78). Interestingly, TSC patients present with differences in disease severity, depending on the underlying mutation, with *TSC2* mutations causing a more severe neurological and cognitive phenotype (22, 79–81). In conjunction with this, *Tsc2*^GFAP^ mice present with more severe epilepsy than *Tsc1*^GFAP^ mice (82).

While the growth advantage of astrocytes plays an apparent role in disruption of neuronal circuits, astrocytes in this model also display functional changes. A pathological hallmark of acquired epilepsy is impaired potassium buffering by astrocytes (83). Its implication in epileptogenesis is based on increased extracellular potassium upon neuronal depolarization, reduced astrocytic clearance of excess potassium, and consequently neuronal hyperexcitability and seizures. Key players in astrocytic potassium buffering represent aquaporins, Kirs, and connexins, which all display dysregulation in TSC-null astrocytes, *Tsc1*^GFAP^ mice, and surgically resected TSC tissue (84–87). Another well-established player in neuronal hyperexcitability is impaired astrocyte-mediated clearance of glutamate, which can predispose neurons to sustained excitability, excitotoxicity, and epileptiform activity. Astrocytes in human TSC display altered glutamate receptor expression, whereas *Tsc1*^GFAP^ mice present with decreased expression of glutamate transporters, implying altered extracellular glutamate metabolism (72, 88, 89). Pharmacological upregulation of glutamate transporters in astrocytes of *Tsc1*^GFAP^ could reduce seizure frequency and some of the pathological changes, exemplifying the likely importance of extracellular glutamate clearance in TSC (88). Lastly, increased astrogliosis and consequent enhanced astrocytic adenosine kinase activity in epilepsy models and various epileptogenic pathologies, including TSC, result in a deficient homeostatic adenosine tone at the synapse and reveal a direct link between astrocyte activation and network excitability (90, 91).

Besides the reported changes in potassium buffering, glutamate clearance, and adenosine homeostasis, TSC is also characterized by inflammatory changes, and astrocytes are supposed to be both source and target of inflammatory signaling therein (92–95). Indeed, human tuber and SEGA tissue also display activation of inflammation in astrocytes, in particular, the toll-like receptor 4 (TLR-4), interleukin 1β (IL-1β), and complement pathways, as well as increased expression of IL-17, intercellular adhesion molecule 1, tumor necrosis factor α (TNF-α), and nuclear factor κB (NF-κB) (61–63, 96–98). In particular, several large-scale RNA-sequencing studies confirmed that neuroinflammation is a hallmark of TSC-associated lesions, and the retrieved data were enriched for both astrocyte and microglial specific genes (21, 63, 99, 100). Additionally, microRNAs (miRNAs) involved in the regulation of astrocytic inflammatory responses are upregulated in TSC (101). In comparison, astrocytes in *Tsc1*^GFAP^ mice also present with increased IL-1β and C-X-C motif chemokine 10 expression, most notably, preceding the development of seizures, and are also characterized by increased microglial proliferation (74). Collectively, proinflammatory changes represent an important pathogenic mechanism by further activating astrocytes and could also reinforce mTOR-related dysfunctional processes, e.g., the immunoproteasome pathway, which might represent a direct molecular link between inflammation, mTOR activation, and epilepsy in TSC and other mTORopathies (102).

An additional pathogenic mechanism frequently encountered and closely linked to inflammation in epilepsy is oxidative stress (OS) (103–105). OS is defined as disturbance in the cell's redox state and was shown to be highly correlated with inflammation in dysmorphic neurons, giant cells, and glia of TSC and other mTORopathies (98). Glial cells in TSC displayed higher expression of the enzymes inducible nitric oxide synthase (iNOS) and cyclooxygenase 2 (COX-2) (98). Both enzymes produce mediators that contribute to OS and inflammation, thereby supporting the notion that glia are mediators of these pathogenic processes in TSC. In addition, giant cells revealed strong expression of OS (iNOS, cysteine/glutamate antiporter) and inflammation (COX-2, TLR-4) markers, as well as accumulation of NF-κB in the nucleus, supporting the strong correlation between these two processes (98). Another article pointed at the critical role of OS promoting an environment that favors the positive selection of cells with higher antioxidant capacity due to aberrant mTOR activation (106). Further research into OS in TSC revealed that the proinflammatory miRNA-155 might contribute to this sustained activation of antioxidant pathways in giant cells and astrocytes, exemplifying the link between OS and brain inflammation (107). Furthermore, the induction of sustained, miR155-mediated antioxidant signaling in astrocytes led to dysregulation of iron metabolism, which could result in the potentiation of OS in TSC (107).

A final pathological hallmark of TSC is the disruption of the blood–brain barrier (BBB) (108), with implications for a modulatory role of matrix metalloproteinases in BBB remodeling (62, 109–113). In this context, chronic BBB dysfunction and epileptogenesis after status epilepticus (SE)–induced epilepsy could be reduced via treatment with rapamycin, pointing toward a more general role of mTOR-dependent BBB remodeling during epileptogenesis in epilepsy (34, 35, 114).

## Giant Cells and Radial Glia

In the context of gliopathy in TSC, it is noteworthy that giant cells represent a cell type with features of immaturity, highlighting the absence of differentiation to macroglial or neuronal lineage cells prior to migration into the developing cortex (72, 115–117). While the exact precursor of giant cells is unclear, the differential expression of glial (GFAP and S100 protein), neuronal (neurofilament, synaptophysin, MAP2), and neuroglial progenitor (SOX2, nestin, vimentin, CD133, β_1_-integrin) markers suggests that these cells reflect intermediary, undifferentiated stages of cellular development (45, 62, 115, 116, 118, 119). Many of the expression changes in astrocytes mentioned before are recapitulated in giant cells in tubers; however, on average, they display high heterogeneity, likely due to variation in the frequency of mutations based on the “two-hit” hypothesis (21, 49). Accordingly, balloon cells in FCD, a cell type histologically resembling giant cells in TSC, have been shown to also carry pathogenic somatic, second-hit variants of mTOR regulatory genes, and their density correlates with genetic findings (120). Moreover, non–cell-autonomous effects of the mutation influencing both the interglial and neuroglial crosstalk during cortical development may also contribute to the histological phenotype of malformed cells. Thus, giant cells and balloon cells might represent an important glioneuronal cell type in the generation of disturbed cellular architecture in developmental malformations related to mTOR dysregulation. Functionally, giant cells might contribute to brain inflammation by expressing complement factors and attracting immune cells already very early in development (62, 121). Moreover, they might be actively involved in the aberrant neuronal circuitry leading to the neurological manifestations of TSC by expressing glutamate and γ-aminobutyric acid (GABA) receptors and transporters (72, 122, 123).

One proposed precursor for giant cells are radial glia, progenitor cells of astrocytes and neocortical neurons, and oligodendrocyte progenitors cells (OPCs) (124, 125). Radial glia are localized in the subventricular zone of the developing brain, giving rise to the proliferative, astrogliogenic/neurogenic niche in the developing brain, as well as providing the physical substrate for neurons to migrate along toward their cortical destination (126). In light of this, radial glia perform vital functions in the formation of the cortex, and their malfunction is hypothesized to give rise to improperly differentiated cells, i.e., giant cells and dysmorphic neurons, and malformed cortical cytoarchitecture. Studies on radial glia-specific *Tsc1* or *Tsc2* knockout mice displayed characteristic features of human TSC, such as aberrant cortical architecture, hippocampal disturbances, astrogliosis, and spontaneous seizures (127–129). Importantly, these alterations displayed specific phenotypic differences between *Tsc1* and *Tsc2* knockout mice. Moreover, organoid model systems revealed that higher mTOR baseline activation in outer radial glia, a feature linked to the stemness of progenitor cells (130), is specific to primate corticogenesis, suggesting that this cell niche is highly susceptible to perturbations due to germline or somatic mutations in the mTOR pathway and thereby could induce aberrant formation of giant cells in the TSC brain (131, 132). This primate-specific feature could also explain the limitations of most TSC model organisms to reflect the histopathological features of TSC, such as tubers and giant cells. The aforementioned studies imply strong phenotypic effects, depending on the timing of the mutation, as well as the cell type affected, potentially explaining the phenotypic heterogeneity of dysmorphic cells and in particular astrocytes in human TSC (27). Another highly intriguing finding from these studies on brain organoid development revealed that cellular subtype differentiation of progenitor cells might be perturbed *in vitro* due to enhanced mTOR-dependent glycolysis and endoplasmic reticulum (ER) stress (132, 133), features also implicated in TSC (134, 135).

## Oligodendrocytes

The central nervous system (CNS) pathology of TSC comprises a range of white matter abnormalities, detectable in presurgical magnetic resonance imaging (MRI) (136, 137), as well as in resected lesional tissue (138). While cortical tubers have classically been the neuropathological hallmark feature observed in these patients, the widespread hypomyelination/dysmyelination has emerged as a synonymous and prominent indication for clinical phenotypes in TSC patients. The cells responsible for the development and maintenance of an intact white matter of the brain are specialized cells called oligodendrocytes. They undergo a complex and precisely timed cycle of proliferation, migration, and differentiation to finally generate myelin by concentrically wrapping axons with multilamellar sheets of plasma membrane consisting of specific proteins and lipids (139). Two distinct terms in regard to white matter pathologies have been established, namely, demyelination and hypomyelination. The term *demyelination* is generally used if there is loss of myelin, occurring after a normal myelin development. This pathology has been studied accurately in patients suffering from multiple sclerosis (27, 140). Hypomyelination, on the other hand, may emerge if myelin production is disturbed or was never initiated, as seen in TSC patients (27).

Recent technological advances in MRI including diffusion tensor imaging (DTI) and fractional anisotropy (FA) have further emphasized hypomyelination in TSC (141, 142). Data revealed that regions involved in the processing of visual auditory and social stimuli contain more dysmyelinated axons in patients, hence supporting behavioral and cognitive characteristics (142). In addition, a major neuropathological aspect is the limited myelination in resected lesions of TSC patients. A recent study has reported a possible involvement of oligodendroglial turnover, indicating that the inhibition of oligodendroglial cell maturation, supposedly due to constitutive activation of mTOR, may lead to insufficient myelination in TSC patients (138). A principal feature of diseases with abnormal white matter is an oligodendroglial pathology that is frequently associated with cognitive impairments (64). The hypothesis that a dysfunctional white matter and hence abnormal neural circuitry account for the neurological manifestations in TSC has been further investigated by a plethora of studies. Interestingly, TSC patients with ASD have more crucial white matter abnormalities compared to patients without ASD (143, 144).

Oligodendroglial development, from an OPC (also called NG2 glia) to the maintenance of an intact myelin sheath, is tightly controlled by a myriad of both extracellular and intracellular factors, with two regulatory pathways in focus: the mitogen-activated protein kinase kinase/extracellular signal-regulated kinases 1 and 2/mitogen-activated protein kinase (Mek/ERK1/2-MAPK), and the mTOR signaling pathway (145, 146). Specifically, during oligodendrocyte lineage progression and initiation of myelination, the mTOR pathway via mTORC1 has emerged as a key player involved in this process (146). In a recent study, the involvement of mTOR signaling in cytoskeletal reorganization during oligodendrocyte development, as well as in initiation of myelination, was demonstrated. Moreover, the importance of the mTOR pathway on oligodendroglial branching complexity was observed (147). One study demonstrated a decrease in both myelin content and oligodendrocytes in and around cortical lesions of mTORopathy specimens (138). This decrease was linked to elevated mTOR expression and a possible impairment of oligodendroglial turnover, suggesting that mTOR pathway mutations cause a defect in oligodendrocytes (138). Thus, high lesional-specific mTOR activation combined with a decreased number of oligodendrocytes may further strengthen the hypothesis of mTOR pathway-dependent modulation of oligodendroglial differentiation and myelination properties. A plethora of studies have proven the essential role of mTOR signaling on the complex differentiation of oligodendrocytes to the maintenance of an intact myelin sheath (148–150).

Animal models have delved further into the causal relationship between mTOR pathway signaling and proper CNS myelin formation and maintenance. However, there is still considerable uncertainty with regard to the cell autonomous effects of TSC ablation in oligodendrocytes or aberrant signaling from TSC-deficient neurons or astrocytes that may indirectly influence myelination processes in the brain. It has now been suggested that CNS myelination, specifically myelin-associated lipogenesis, and protein gene regulation are mainly dependent on mTORC1 function (151). The same authors demonstrated that oligodendrocyte-specific enhancement of mTORC1, via loss of TSC1, results in abnormal myelination in mice (151). Remarkably, brains of *Tsc2*^Olig2^ KO mice reveal distinct hypomyelination, further supporting a cell-autonomous effect of TSC2 inactivation on oligodendrocytes (152). Grier et al. (153) drew attention to the important but more transient contribution of mTORC2 signaling in myelinogenesis by utilizing a mouse model lacking the rapamycin-insensitive companion of mTOR (Rictor), a functional component of the mTORC2, in NG2 glia. They were able to observe that loss of Rictor in these cells decreases and delays the expression of myelin related proteins and causes a developmental hypomyelination (153).

Besides cell-autonomous effects, a recent study supports the role of abnormal neuron–oligodendroglia communication causing hypomyelination employing induced pluripotent stem cell–derived neuronal and oligodendroglial cultures from TSC patients. Interestingly, neuron–oligodendrocyte cocultures from these patients revealed increased oligodendrocyte proliferation but a decrease in maturation (154). In terms of neuron–glia interaction, it was shown that *Tsc1* mutant mice display a striking delay in myelination supporting the hypothesis of an underlying abnormal neuron–oligodendrocyte communication that causes white matter pathologies (155). Further, neuron-specific ablation of *Tsc1* in a mouse model results in an increase in connective tissue growth factor secretion that leads to a decrease in the number of oligodendrocytes (156).

In conclusion, there is evidence that mTOR signaling is indeed fundamental to oligodendrocyte differentiation and myelination, as well as critical indications that both cell-autonomous effects and interactions between neurons and oligodendrocytes cause hypomyelination in mTORopathies. Because the outcome of the mTOR pathway hyperactivation observed in TSC patients as well as in animal models is hypomyelination and not the expected hypermyelination, at least five mechanisms were hypothesized to be responsible for this paradoxical impact on myelinogenesis. The constitutive mTORC1 signaling might account for (1) a delayed onset of myelination, (2) triggering non-physiological toxic effects, such as ER stress or apoptosis of oligodendrocytes, (3) TSC subunits exerting non-canonical functions that are independent of mTORC1, (4) suppressing mTORC2 functions, and (5) a negative feedback on mTORC1-independent targets, such as Mek-Erk 1/2 and/or PI3K-Akt pathways [for a detailed review, see (157)].

Because of the emerging evidence for a link between decreased myelin content and the development of neurological deficits, achieving remyelination of axons takes center stage in multiple sclerosis research, implying that it might be beneficial for mTORopathy patients as well (158, 159). As far as disease control is concerned, an important question is whether the observed hypomyelination in TSC patients may be reversible by reducing the constitutive activation of the mTOR signaling pathway. Latest research emphasizes the use of rapamycin or the rapamycin analog, everolimus. Only few researchers have addressed the question if and how the white matter is altered after treatment with an mTOR inhibitor. A pharmacological therapy administering everolimus was able to decrease mean diffusivity and increase FA during DTI measurements in TSC patients (160). In terms of everolimus treatment period, recent data support the hypothesis that longer periods improve the white matter microstructural integrity even more (161). In summary, evidence from experimental and human studies indicates that hypomyelination could be reversed by treatment with everolimus; however, the mechanism of action needs to be studied in more detail.

## NG2 Glia

Apart from astrocytes and oligodendrocytes, NG2 glial cells represent a third macroglial subtype in the CNS, which has received much attention in the past decade [for a detailed review, see (162)]. In the literature, these cells are primarily referred to as OPCs, but also as complex cells, synantocytes, polydendrocytes, and GluR cells, as they depict glial and neuronal functions (163, 164). NG2 glial cells are substantially spread in both gray and white matter of the developing as well as the adult brain (165, 166). A remarkable feature found in cells expressing the proteoglycan NG2 is their proliferative and differentiation potential throughout life (166, 167). Interestingly, in post-natal white matter, these cells mainly differentiate into myelinating oligodendrocytes (168–170), and especially following demyelination, this process is amplified (171). In contrast, NG2 glia in the gray matter retain their neuronal–glial properties post-natally (11). These cell populations receive direct neuronal glutamatergic and GABAergic synaptic input and express voltage-gated ion channels for K^+^, Na^+^, and Ca^2+^ that can trigger long-term potentiation; however, they do not generate action potentials themselves (172–175). The precise functional changes of these cells in response to synaptic input remain largely unknown except some evidence on modulation of inward rectifying potassium channels (176) and proliferation (177, 178). Interestingly, cleaved NG2 was shown to rescue diminished neuronal α-amino-3-hydroxy-5-methyl-4-isoxazolepropionic acid (AMPA) currents in NG2 knockout mice, establishing a reciprocal signaling loop between NG2 and neurons (179). Apart from these neuronal properties, NG2 glia in the human hippocampus do not couple to other glia via gap junctions, such as astrocytes and lack glutamate transporters, while expressing Kirs (180). Moreover, NG2 ablation was shown to induce microglia-mediated neuroinflammation and neuronal death in the hippocampus. The authors concluded that reduced NG2-derived trophic support via hepatocyte growth factor was responsible for this loss of neurons (181). Furthermore, NG2-derived transforming growth factor β2 (TGF-β2) signaling to TGF-β receptor 2 on microglia was shown to be a key regulator of microglial CX3CR1-mediated immune responses, and deficiency of this signaling axis via NG2 ablation led to neuronal loss and inflammation (182). Hence, the ability of NG2 glia to respond to neuronal inputs, as well as retaining a high proliferative potential in the human brain, makes this cell type another interesting glial cell in the context of TSC.

So far, only one study directly characterized NG2 cells in TSC tubers (52). Although these authors concluded that there were no detectable morphological alterations in oligodendrocytes and NG2 cells, they also acknowledged the lack of knowledge concerning specific activation markers for these cell types. Moreover, this study mainly evaluated morphological changes from an astrocyte perspective and did not convincingly rule out functional changes in NG2 glia (52). Because mTOR is an essential regulator of oligodendrocyte differentiation during development (157), its therapeutic potential was investigated by several studies. Moreover, the OPC-specific NG2 proteoglycan appears be directly linked to mTOR and to positively regulate its activity (183). Deletion of either *TSC1* or phosphatase and tensin homolog (*PTEN*) in NG2 cells, both negatively regulating mTOR, induced an increase in mTORC1 activity. Interestingly, whereas *TSC1* deletion in these cells led to the expected hypomyelination and impaired oligodendrocyte development, ablation of *PTEN* resulted in enhanced NG2 glia proliferation and oligodendrocyte lineage progression. This suggests the involvement of an mTORC1-independent PTEN-downstream signaling process. Further, also deletion of the PTEN-AKT downstream target glycogen synthase kinase 3β (*GSK3ß*) resulted in a comparable increase in differentiation of oligodendrocytes (184). These results may indicate a possible remyelination mechanism via inhibition of the PTEN-AKT-GSK3β pathway. McLane et al. (185) further revealed that an ablation of *TSC1* affects oligodendroglia differently, depending on the olig odendroglial lineage stage. A deletion of *TSC1* from NG2 glia speeds up the remyelination process, although *TSC1*-deficient proteolipid protein–positive oligodendrocytes decelerate remyelination.

Although most research on NG2 glia focused on their function as OPCs, there are also emerging lines of evidence that link them to neuronal function and microglia-mediated neuroinflammation. In the context of TSC, it would be interesting to study models of mTOR activation specifically in gray matter NG2 cells, where they reportedly serve more diverse functions.

## Microglia

Opposed to other neuroglia that are brain-borne, microglia arise from yolk sac–primitive macrophages and invade the brain during development before the BBB is fully formed (186–190). This early migration is a well-preserved mechanism among species, thereby emphasizing the important role of microglia during brain development (188, 191–193). Indeed, the phagocytic function of microglia is most prominent during development as they are capable of phagocytosing newly formed neurons and synapses in the developing brain (194–196). In the adult brain, ramified microglia surveil the brain environment with their processes and migrate to areas of need in response to activation cues, such as chemokine signaling (197). In response to distress signals, microglia can become activated, which is accompanied by a variety of morphological and molecular changes (198, 199). In general, two states of activation can be distinguished: a proinflammatory state (classically M1) that allows immune responses against pathogens and dysfunctional cells, which at the same time might exert damage on surrounding healthy tissue; and an anti-inflammatory state (classically M2) that is thought to be central in repair processes (200–202). However, thanks to sequencing data, microglia activation was identified to be a continuum in which many subtypes can be distinguished (203–206). In addition to their classical role as resident immune-competent cells and noteworthy in the context of TSC, microglia were also shown to modulate neuronal activity directly (207) and can be activated in response to excessive neuronal activity in epilepsy (208).

Several studies have shown increased density and activation of microglia cells in the brains of TSC patients (45, 62, 110, 121, 209). In cortical tubers, microglia with an activated morphology were found throughout the lesional tissue, mostly localized in close proximity to dysmorphic neurons and giant cells with mTOR activation, indicated by phosphorylation of the mTOR target ribosomal protein S6 kinase (pS6K), as well as around blood vessels (62). Similarly, in other epilepsies characterized by mTOR activation, such as FCD, TLE, and Rasmussen encephalitis, increased expression of microglial markers has been found in the respective brain lesions (33, 209–213). Moreover, in TLE patients with glial scarring due to drug-resistant epilepsy, mTOR activation was mostly found in microglia and to a lesser extent in astrocytes (33). Functionally, these microglia have been suggested to have a damaging role as they were shown to colocalize with several proinflammatory markers and surrounded cells expressing caspase 3, indicating that they might be involved in apoptosis (62). Moreover, in FCD lesions and glioneuronal tumors, the number of microglia has been correlated with seizure frequency of the patients (210, 214). Although it remains difficult to pinpoint whether microglia activation is causative or consequential of neurological deficits in these pathologies, the colocalization with pS6-positive cells indicates that microglia respond to mTOR hyperactivation in TSC. Sun et al. (215) showed that microglia activation in FCD and TSC might be partially caused by reductions in the immune modulatory molecules CD47 and CD200 on neurons and their respective receptors on microglia. When exogenously introduced, these molecules could potentially exert anti-inflammatory effects on microglia by suppressing proinflammatory cytokines, such as IL-6 (216).

Several attempts have been made to study aberrant mTOR activation in microglia and its impact on their function. Zhao et al. (217) showed that deletion of the *Tsc1* gene in CX3CR1-expressing cells (referred to as *Tsc1*^CX3CR1^), either congenitally or post-natally, increased microglial mTOR activity and their overall number in the cortex and hippocampus. All of the *Tsc1*^CX3CR1^ mice developed spontaneous recurrent seizures around 5 weeks of age, as well as two-thirds of the post-natally induced conditional knockout mice. However, that same year, Zhang et al. (218) reported that CX3CR1-Cre driver lines in *Tsc1*^CX3CR1^ animals target not solely the alleged microglia cells, but also NeuN- and rarely GFAP-positive cells. Therefore, they concluded that the effects seen in *Tsc1*^CX3CR1^ mice were not exclusively driven by reactive microglia but could also be elicited by affected neurons and astrocytes. Furthermore, post-natal induction of the knockout, which had a higher specificity for microglia showing only 5% of non-microglial cells affected, did not result in spontaneous ictal activity (218), in contrast to the previous study (217). These studies emphasize that it is essential to precisely target and characterize cell types in TSC KO models, as only small percentages of affected neurons can lead to increased neuronal excitability (219). Nevertheless, isolated microglia from *Tsc1*^CX3CR1^ animals displayed clear cellular alterations. Thus, these two studies support the role of mTOR-dependent microglial abnormalities, and its role in epileptogenesis, especially in the context of inflammation, cannot be excluded.

In another model, direct manipulation of the mTOR pathway was induced by *in utero* electroporation of constitutively active Rheb, an mTORC1 activator. With this method, Nguyen et al. (73) observed that mTOR hyperactivity resulted in a global increase in Iba1-positive microglia that were both larger and had a more activated morphology. Furthermore, these Iba1-positive microglia were positively correlated with seizure frequency. However, with this technique, not only microglial cells were targeted. Indeed, mTOR activation also induced hypertrophy and cytoarchitectural misplacement of neurons in these animals, which together with the activated microglia were concluded to be responsible for seizure generation. Finally, in the BV2 microglial cell line direct activation of mTOR by MHY1485 treatment *in vitro* induced expression of proinflammatory cytokines, such as TNF-α, IL-6, and HMGB1, and decreased anti-inflammatory cytokines, such as TGF-β and IL-10. Furthermore, microglia displayed a shift from an anti-inflammatory toward a proinflammatory subtype, and markers of autophagy were reduced due to mTOR activation (220).

Besides direct genetic or chemical activation of the mTOR signaling pathway, the majority of research on the interaction of microglia and mTOR is supported by experiments that evaluated microglia in disease states characterized by increased mTOR activity through various brain injuries and/or by means of chemical mTOR inhibition. For example, pilocarpine-induced SE resulted in mTOR activation in neurons and microglia, and subsequent rapamycin treatment could alleviate microgliosis and had beneficial effects on cognitive performance of animals (221). Moreover, kainic acid–injected rats treated with rapamycin displayed reduced microglial activation (35). In contrast, rapamycin treatment in an electrically induced post-SE model did not change expression of inflammation markers or number of CD11b/c and CD68-positive cells, indicating that rapamycin did not affect brain inflammation in this model. Other studies have used brain injuries, such as stroke or vascular dementia in combination with mTOR blockage to evaluate microglial changes. Treatment with rapamycin or its derivatives everolimus or sirolimus could revert medial cerebral artery occlusion (MCAO)–induced increases of cytokines and/or chemokines, as well as promote anti-inflammatory microglial polarization (222, 223). In this same study, Raptor^CX3CR1^ mice, characterized by lacking regulatory-associated protein of mTOR (Raptor) specifically in microglia leading to mTORC1 activation, undergoing MCAO were found to have similar beneficial responses to chemical mTOR inhibition in terms of microglia activation and cytokine induction. Treatment with everolimus in mice with bilateral common carotid artery stenosis, a vascular dementia model that induces mTOR activation, also caused a shift toward anti-inflammatory microglia due to a loss of inhibitory feedback of mTORC1 on PI3K, alternatively activating the prosurvival kinase Akt (224). Likewise, spinal cord injury induced increases in OX42-positive microglia, which could be attenuated by treatment with wortmannin, an inhibitor of the PI3K/Akt/mTOR pathway (225). Interestingly, according to Yang et al. (226), because of its ability to also interact with mTORC2, everolimus is more effective than rapamycin in counteracting lipopolysaccharide (LPS)/kainic acid–induced microglial responses. Of note, some authors argued that the anti-inflammatory effect of mTOR inhibitors might be mediated primarily by other cell types than microglia (222). Despite these claims, in pure microglia cell cultures, such as the BV2 and N9 cell line, inhibition of mTOR activity after oxygen glucose deprivation, LPS, or a cytokine challenge reduced both microglia activation and inflammation (224, 227–229). Furthermore, LPS-stimulated N9 microglia even exerted neuroprotective effects after rapamycin treatment, as conditioned medium could suppress neurotoxicity in a neuronal cell line (229). Lastly, in a kainic acid–induced SE model, the long-term epileptogenic effects of early life seizures could be reduced via treatment with an inhibitor of microglia activation, minocycline, directly linking microglial activation and epileptogenesis (230).

Finally, assuming microglial activation secondary to mTOR-driven malformations of cortical development in TSC, depletion of resident microglia, and repopulation of the brain with fresh microglia might offer a drastic, yet promising therapeutic option to resolve chronic neuroinflammation (231). Importantly, this approach could relieve the neuroinflammatory burden in the post-natal TSC brain even after aberrant brain development. Adjunctive with mTOR inhibitors, this approach could target source (mTOR hyperactivation) and symptom (microglia activation) of TSC brain malformations simultaneously and offer a valuable disease-modifying therapy.

## Interglial Crosstalk

**Figure 2:**
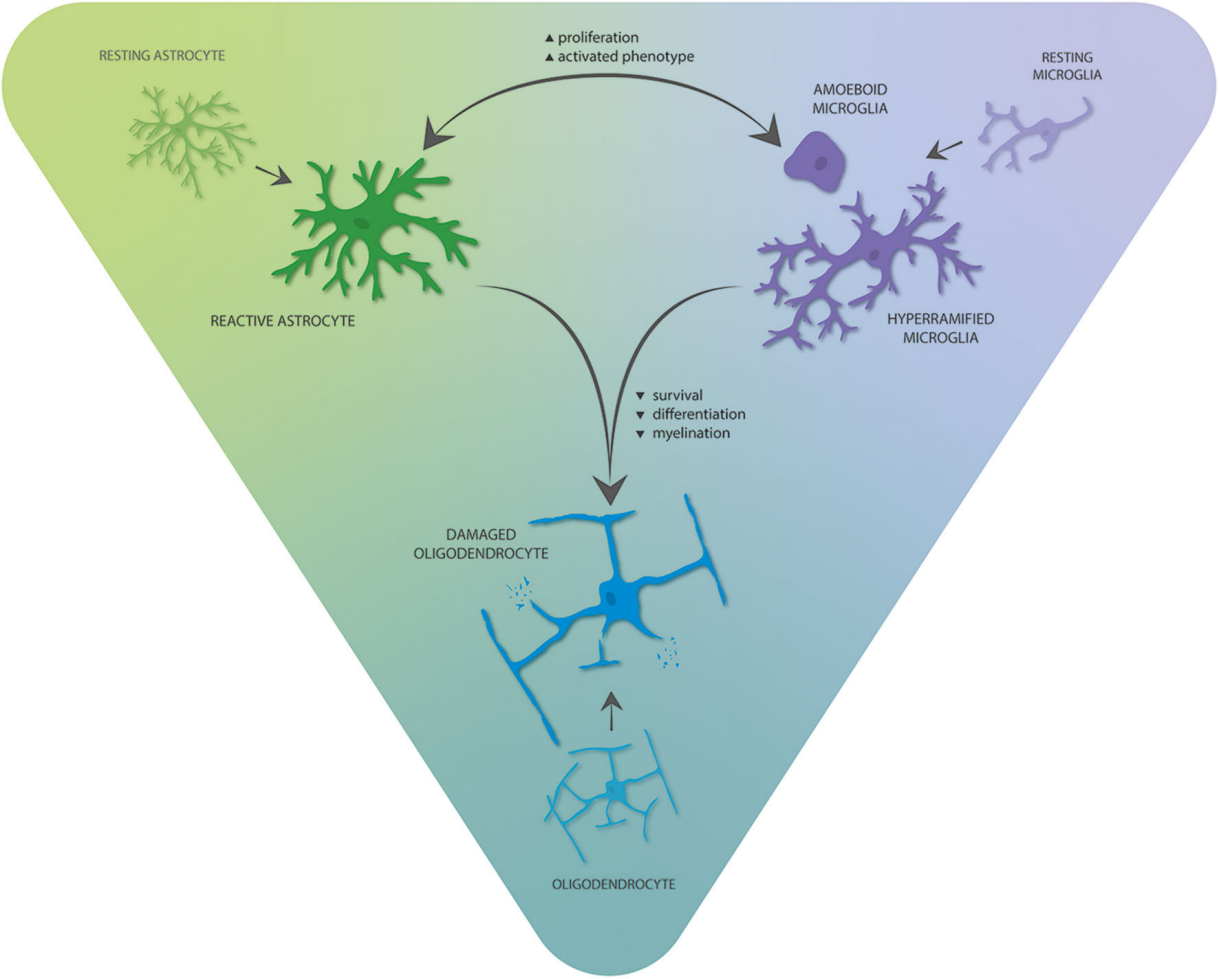
Interglial crosstalk of the three main glial cell types in the TSC brain. Astrocytes and microglia can stimulate and reinforce proliferation and phenotypic activation of each other, thereby promoting proinflammatory gene expression. These alterations mediate negative consequences on oligodendrocyte survival, differentiation, and myelination.

While neuron–glia interactions are the focus in many of the studies discussed here, interactions between glia may offer new therapeutic and diagnostic opportunities (Figure 2). In the context of neuroinflammation, bilateral signaling between microglia and astrocytes likely plays an essential role in brains of TSC patients. For example, *Tsc1*^GFAP^ mice do not only display alterations in astrocytes, but microglia number and size were abnormally increased in cortex and hippocampus, pointing toward an indirect effect of mTOR hyperactivation in astrocytes on microglia (232). However, the importance of microglia in the induction of a reactive phenotype in astrocytes has been shown (233), and *Tsc1*^CX3CR1^ mice also display increased proliferation and reactive changes of astrocytes (217). Together, this reinforcing crosstalk might be crucial for the maintenance of a proinflammatory environment in TSC with contributions from functionally normal glia, as well as glia with cell-autonomous mTOR-related alterations. The effect of microglia is likely contributing to the proinflammatory environment of TSC tubers as their functions involve inflammation initiation and propagation in conjunction with astrocytes (233, 234). Moreover, microglia activation may exert proinflammatory/damaging effects on oligodendrocytes and neurons, contributing to neuronal dysfunction and resulting neurological comorbidities and hyperexcitability (235, 236).

Next to microglia, particularly interesting in the context of interglial crosstalk in TSC is the role of astrocytes to directly influence the production and survival of cells of the oligodendrocyte lineage (237, 238). Accordingly, reactive and enlarged dysplastic astrocytes with enhanced activation of mTOR and gain of aberrant functions in cortical tubers, including a proinflammatory phenotype, may pose detrimental in the function of other glia in TSC. In support of this, there is a growing body of evidence that supports the concept of astrocytopathies within the field of childhood white matter disorders in which dysfunctional astrocytes have been suggested to drive degeneration of the white matter (239, 240). As mentioned previously, astrocytic gap-junction coupling in TSC models is disturbed (85), and heterotypic gap-junction coupling between astrocytes and oligodendrocytes was shown to be essential for (re-)myelination in animal models (241–243). Moreover, dysregulation of glutamate metabolism by astrocytes in TSC (72, 88, 89) could promote excitotoxic cell death in oligodendrocytes as they express functional *N*-methyl-d-aspartic acid, AMPA, and kainate-type receptors that mediate toxic effects of excess glutamate (244–247). Moreover, astrocyte-specific NF-κB activation in TSC might also play a role in suppressing myelination (248). Lastly, evidence from the “twitcher” mouse model supports the role of microglial COX-2 in demyelination. Here, secreted microglial prostaglandins (PGDs) could stimulate PGD receptors on astrocytes, inducing astrogliosis as indicated by hypertrophy and a rise in intracellular calcium, and blocking this pathway increased oligodendrocyte survival (249). Because increased COX-2 expression is observed not only in glia, but also giant cells/balloon cells and dysmorphic neurons in TSC and FCD (98), this specific crosstalk might link hypomyelination to COX-2 expression.

As for mature oligodendrocytes, NG2 function in TSC likely depends on other glia. *In vitro*, it was shown that astrocyte- and microglia-conditioned medium exerts important effects in the development of oligodendrocytes, with astrocytic factors promoting oligodendrocyte survival and microglial factors supporting differentiation and myelination (250). Considering aberrant number and function of both cell types already early in development, this interglial crosstalk might contribute to the hypomyelination observed in TSC. Moreover, NG2 glia survival and differentiation can be impaired by OS and TNF produced by activated microglia (251–253). In essence, astrocytes and microglia could participate in the pathological link between OS, inflammation, and the dysregulated iron metabolism in TSC by inducing aberrant oligodendrocyte maturation and myelination in TSC (252, 254). Importantly, OS-dependent dysregulation of histone–deacetylase activity could promote astrogenesis/neurogenesis over oligodendrogenesis potentially contributing to the disturbed cell ratio observed in TSC brain tissue (252).

## Conclusions

While the most debilitating CNS symptoms of TSC, epilepsy, and neurodevelopmental comorbidities ultimately result from neuronal dysfunction, it is also clear that glial alterations contribute and shape the complex mechanisms generating these symptoms. Moreover, glia provide the proliferative precursor of pre-natal and post-natal neurons in the form of radial glia and astrocytes, respectively. It is important to stress that in TSC there is likely a mixture of cells with cell-autonomous mTOR activation because of intrinsic TSC mutations and cells with regular mTOR activity that respond to changes due to this intrinsically dysfunctional cellular substrate. Nevertheless, the major triad of glial cells displays conserved features in response to mTOR activation in TSC, TSC models, and conditions of mTOR hyperactivation.

Although it is likely that increased proliferation of astrocytes and resulting physical disruption of neuronal circuits can impact epileptogenesis in TSC, studies on surgically resected tubers and TSC models suggest that astrocytes also present with epileptogenic functional changes. More importantly, these changes are likely caused by a mixture of primary astrocytic changes during brain development due to mTOR activation and secondary effects that promote reactive states of astrocytes, such as brain inflammation later on. Finally, astrocyte dysfunction in TSC recapitulates findings from other epileptogenic pathologies, thus potentially representing common astrocytopathic mechanisms of epilepsy that could be targeted by novel astrocyte-centered therapies.

For oligodendrocytes, it is of utmost interest to find targets by which the endogenous remyelination in TSC patients might be enhanced. The mTOR signaling pathway has been proposed to be an attractive target to promote remyelination; however, recent results emphasize the importance of correctly applied therapeutics, because what may be beneficial to OPC development might be noxious to myelinating oligodendrocytes.

Lastly, alterations in microglial functions in TSC might be caused by cell-autonomous mTOR activation or secondary to the altered brain environment in TSC. Whether their activation depends on either or both is not clearly defined yet; however, the presence of proinflammatory microglia upon mTOR activation likely contributes to pathology, while a shift toward an anti-inflammatory phenotype via mTOR inhibition might have beneficial effects.

Although challenging, a better understanding of the complexity of the glial pathology in TSC may provide opportunities for novel therapeutic approaches targeting glial-mediated mechanisms. In particular, a combinatorial therapy targeting different glial cell types and their crosstalk might be translated into disease-modifying treatments for various epilepsies associated with a deregulation of mTOR. Considering the evidence for mTOR inhibition not only rescuing neuronal, but also glial dysfunction, in preclinical TSC models (29, 255, 256), mTOR inhibitors, which were recently approved by the US Food and Drug Administration and European Medicines Agency for the treatment of refractory seizures associated to TSC starting from the age of 2 years (257), represent promising candidates to target TSC gliopathy. Finally and most importantly, mTOR inhibition as therapy of TSC could potentially be extrapolated to other genetic and acquired epilepsies (258, 259).

## Author Contributions

TZ and EA drafted the content of this review. TZ, DB, VG, EV, and AM contributed the specific topics and assisted in the final editing and revision of the manuscript. EA conceived the idea and was invited to participate in the editorial issue. All authors contributed to the article and approved the submitted version.

## Conflict of Interest

The authors declare that the research was conducted in the absence of any commercial or financial relationships that could be construed as a potential conflict of interest.
